# The Foreign Journals: Midwifery, and Diseases of Women and Children

**Published:** 1842-10

**Authors:** 


### MIDWIFERY AND DISEASES OF WOMEN AND CHILDREN.

### *On the Management of Cases of Prolapsus of the Funis.* By Professor OSIANDER, of GÖTTINGEN.

AFTER a brief historical sketch of the various plans which have been adopted for the management of this accident, Professor Osiander relates several cases in illustration of the method of treatment to which he gives the preference. He sums up with the following rules, in estimating which, however, the partiality of the Professor for the long forceps must not be forgotten. He observes that the possession of a pair of forceps at least sixteen inches long is indispensable to the successful practice of midwifery. Professor Osiander's conclusions are:

1. Manual intervention is not required in every case of prolapse of the funis beyond the os uteri. It very frequently happens that the head passes beyond the funis, and that labour is terminated without any accident, though the case is left entirely to nature.

2. When the conditions are favorable, that is to say when the child is of moderate size, when the structure of the parts is natural, and the pains are effective, it is best to leave the case entirely to nature. Moderate pressure on the cord is seldom dangerous in these cases any more than when the funis is twisted round the child. At the most, if the process of labour is slow it may be proper to apply the forceps.

3. Turning should not be resorted to unless some other circumstance than the prolapse of the cord renders it necessary. The old axiom that in all cases of funis presentation the child is to be turned is as ill-founded as it is mischievous.

4. Cessation of pulsation in the cord is not a certain sign of the death of the fœtus, and is rather an indication for hastening delivery than a reason for neglecting the condition of the child.

5. Attempts to replace the prolapsed funis within the uterus are seldom indicated; but on the contrary are almost always fruitless, while they are likely to interrupt and arrest the process of labour. If, however, the funis is low down in the vagina, or has descended out of it, it must be replaced, and retained within it by a sponge, a compress, or other means, since the action of the cold air speedily interrupts the circulation in the cord and occasions the death of the fœtus.

*Neue Zeitschrift für Geburtskunde.* Band xii. Heft i.

---

*On the employment of Preparations of Quinine in the Diseases of Children.* By M. TROUSSEAU.

In a lecture delivered by him at the Hôpital Necker, M. Trousseau made some observations on the difficulty of administering the sulphate of quinine to children, owing to its nauseously-bitter taste. In reference to this he recommends the impure quinine (quinine brute), which is more active than the sulphate of quinine, is insoluble in the saliva, and has not much taste. One hundred parts of it contain sixty-five of pure quinine, thirty of cinchonine, and only five of water; while in one hundred parts of the sulphate of quinine there are thirty of water. This preparation is soluble in lactic acid, and is quickly changed into soluble salts in the stomach by the acids which exist in that viscus. Its resinous consistence is another advantage, since it admits of its being rolled into pills; in which form it may be mixed with sago, or given in any vehicle which contains no acids, since they would at once convert it into bitter and insoluble salts.

*La Clinique des Hôpitaux des Enfans. Mai* 15, 1842.

---

*Account of a case of extra-uterine Pregnancy, the subject of which had attained her seventieth year.* By M. J. AUBRY.

On September 25, 1841, a female was admitted into the hospital Cochin, under the care of M. Blache. She had given birth to one child at the age of 27 years, and had ceased to menstruate at fifty. She had always had good health till she was 40 years old, but then she became subject to dull pains which returned at irregular intervals and were referred to the left side and iliac fossa. After these pains had lasted for some years a prominence appeared at the left side and lower part of the abdomen, and for the last ten years the pain had been more severe, obstinate constipation had supervened, and the patient had grown very thin. At the date of her admission into the hospital she was in a state of exhaustion, so great that for several months she had kept her bed. Her abdomen was tender, tense, and tympanitic, and in the left iliac fossa was a large, hard, and irregular tumour, which was taken for an ovarian cyst. The patient died on the day of her admission.

On examining the body after death, signs of recent peritonitis with serous effusion mixed with shreds of lymph were found in the abdominal cavity. The great omentum was adherent to the borders of the left iliac fossa and covered the tumour, which occupied almost the whole of the cavity of the pelvis, and compressed both the rectum and bladder. In front of the tumour was a portion of the sigmoid flexure of the colon, in which no perforation could be perceived, but its parietes presented considerable friability. The uterus was situated at the lower part and right side of the tumour, which resembled an enlarged ovary. None of the uterine appendages could be distinguished. The interior of the tumour contained the remains of a fœtus, all whose bones were connected together by imperfect ligaments which possessed very little firmness. The skeleton was rolled on itself in such a manner that the lower limbs occupied the posterior part of the tumour, while the occiput corresponded to its anterior part. More than two thirds of the mass were formed by the head, the bones of which overlapped each other, but formed a cavity containing a moderately firm white mass. The bones of the head were hard, and did not present any traces of cartilage, but they differed much from the form of fully developed bones. The articular extremities of the long bones were not formed, but they resembled cylinders somewhat enlarged at either end. The thigh bones were five centimetres and three millimetres, or about two inches long, the tibiæ four and a half centimetres. Very little of the soft parts remained, and, with the exception of a small portion of aorta, and a few pieces which presented a fibrous or membranous appearance, they formed a soft shapeless mass, in which no organ could be recognized.

The seat of the ovum in this case could not be ascertained, since the appendages of the uterus were not distinguishable. The age of the fœtus is another point which cannot be ascertained with precision, since, though the bones were fully ossified, their form differed from that which they present at the full term of utero-gestation. Their anomalous form was probably due to the cartilage which had originally covered them having been absorbed. No reason could be assigned for the occurrence of peritonitis after the abdomen had tolerated the presence of the cyst and its contents for so many years.

*Archives Génèrales de Médecine. March,* 1842.

---

*Singular case.* By Dr. Russegger.

A woman, who had already borne four healthy children, was, in the seventh month of her fifth pregnancy, bitten in the right calf by a dog. The author saw the wounds made by the animal's teeth; which wounds consisted of three small triangular depressions; by two of which the skin was merely slightly ruffled; a slight appearance of blood was perceptible in the third. The woman was, at the moment of the accident, somewhat alarmed; but neither then nor afterwards had any fears that her fœtus would be affected by the occurrence.

Ten weeks after she had been bitten, the woman bore a healthy child, which, however, to the surprise of every person, had three marks corresponding in size and appearance to those caused by the dog's teeth in the mother's leg, and consisting, like these, of one larger and two smaller impressions. The two latter which were pale, disappeared in five weeks; the larger one also is not now so large or deep-coloured as it was at birth. The child is, at present, four months old. *Oesterreich. Medicinische Wochenschrift.* No. 19, *Mai* 7, 1842.

